# Users’ acceptance of electronic patient portals in Lebanon

**DOI:** 10.1186/s12911-020-1047-x

**Published:** 2020-02-17

**Authors:** Gladys N. Honein-AbouHaidar, Jumana Antoun, Karim Badr, Sani Hlais, Houry Nazaretian

**Affiliations:** 1 Hariri School of Nursing, American University of Beirut, Riad El-Solh, Beirut, 1107-2020 Lebanon; 2 Faculty of Medicine, Family Medicine Department, American University of Beirut, Riad El-Solh, Beirut, 1107-2020 Lebanon; 3 Faculty of Medicine, Saint- Joseph University of Beirut, Beirut, Lebanon; 4 American University of Beirut Medical Center, American University of Beirut, Riad El-Solh, Beirut, 1107-2020 Lebanon

**Keywords:** Patient portal, Electronic health record, Middle East, Arab, User acceptance, TAM model

## Abstract

**Background:**

Acceptance of Electronic patient portal (EPP) is instrumental for its success. Studies on users’ acceptance in the Middle East region are scarce. This study aims to use the TAM as a framework to quantitatively describe potential users, diabetic and chronic high blood pressure patients and their providers, intention to use and factors influencing the intention to use EPP at AUBMC-FMC We concurrently test the internal construct validity and the reliability of the TAM.

**Methods:**

A cross-sectional survey design and the vignette approach were used. For validation, we needed a minimum of 180 patients; all 35 attending physicians and 11 registered nurses were targeted. We used descriptive statistics to calculate the intention to use EPP and its determinants based on the TAM constructs. Exploratory factor analysis (EFA) and structural equation modeling (SEM) were employed to estimate significant path coefficients for patients only as the sample size of providers was too small.

**Results:**

We had 199 patients, half intended to use EPP; 73% of providers (*N* = 17) intended to use EPP. Perceived ease of use and privacy concerns were significantly higher among providers than patients (Mean (M) = 0.77 vs M = 0.42 (CI: − 0.623; − 0.081)) and (M = 3.67 vs M = 2.13, CI: − 2.16; − 0.91) respectively; other constructs were not significantly different. Reliability of TAM revealed a Cronbach Alpha of *α*=.91. EFA showed that three components explained 73.48% of the variance: Behavioral Intention of Use (14.9%), Perceived Ease of Use (50.74%), Perceived Usefulness (7.84%). SEM found that perceived ease of use increased perceived usefulness (standardized regression weight = 0.49); perceived usefulness (0.51) had more predictive value than perceived ease of use (0.27) to explain the behavioral intention of use of the EPP.

**Conclusions:**

We found that providers valued the usefulness of EPP and were mostly intending to use it. This finding has yet to be tested in future studies testing actual use as intention and actual use may not be concordant. The intention to use among patients was lower than those reported in developed countries. We identified two factors that we need to address to increase use, namely perceived ease and usefulness, and proposed practical implications to address them; future research directions were also discussed.

## Background

Electronic technology (ET) permeates various aspects of our modern society. Many use the Internet for shopping, social networking, banking [1, 2], and for seeking health information [3–5]. Some use mobile applications or web portals to support behavior modification such as physical activity, diet control, and smoking cessation [6–8].

Electronic Patient Portals (EPPs) are one form of ET pervading the health care system. EPPs are a secure online platform where patients enter a password to access their clinical summaries as well as a spectrum of communication features enabling them to communicate directly with their providers at any time and from anywhere. The impact of EPPs on health care is well documented. Studies show the use of EPPs mitigate difficulties booking appointments and renewing medications [9, 10], and facilitate patient-provider communication [11–13]. EPPs improve patients’ self-care by assisting them in making lifestyle changes and improving their engagement in health promotion and health prevention activities [13–16].

For patients with chronic diseases, EPPs improve treatment adherence and clinical outcomes [17–21]. EPP features that allow patients to record, edit and retrieve their health care data such as blood pressure, blood glucose and weight enable patients to monitor their health and to early detect critical situations and timely intervention [22, 23]. The ability to view their own clinical summaries (problem and medication list), increase patient awareness of important aspects of their own diseases and enable the health care team to identify gaps in self-management to target them with health education [24]. EPP use was also associated with improved medication adherence and controlled blood pressure [25–27].

EPPs are of little value unless they are used meaningfully. Several factors can influence the meaningful use of EPPs, including interface with the technology, individual characteristics of users, and acceptance of the technology. Interface with EPP factors includes the language used to communicate with the provider and self-efficacy in using technology [19, 21, 28–33]. Users’ characteristics, such as health literacy, age, ethnicity, and cultural factors, influence the meaningful use of EPP [19, 28, 30, 31, 34, 35]. A plethora of studies has found that users’ acceptance is the main lever for meaningful use of EPP and a critical factor in determining its success or failure [36–40]. Acceptance is defined as a process that begins with users’ intention to employ the technology, followed by actual use, and if found efficient and effective, then they accept and adopt the technology [39].

**Fig. 1:**
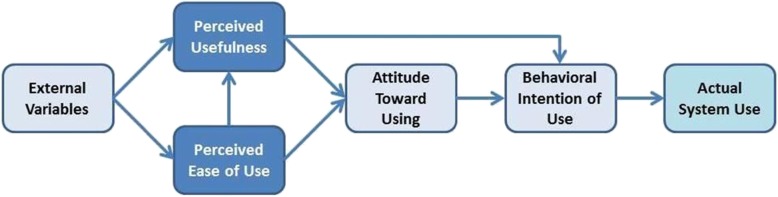
The Technology Acceptance Model (Version 1) [37]

Because acceptance of technology has salient theoretical and practical implications, many researchers have proposed theories or models to predict users’ acceptance of technology. There are about fourteen theories or models focusing on users’ acceptance of the technology [41]. The main objective of those theories or models is to identify barriers for adoption to promote the use of technology [41]. In this study, we opted to use the Technology Acceptance Model [38] version 1 [42] (Fig. 1). TAM is one of the most popular models that focuses on psychological factors influencing acceptance. The TAM measures acceptance in terms of reported intention to use and subsequent technology usage. This framework posits that perceived ease of use of the technology, perceived usefulness of the tasks to be performed, external factors, and attitude predict acceptance and adoption [42]. This model was selected for this study due to its relatively high explanatory power of predicting a broad range of factors influencing intention to use (R^2^ = 0.52); TAM posits an association between perceived usefulness and perceived ease of use, not reflected in other models [43, 44] and its parsimony (few predictors) [45].

To our knowledge, the reliability and validity of this model in predicting use have not been tested in the Arab world thus it is important to examine whether the constructs of the TAM have high explanatory power for predicting factors influencing use in this specific culture.

### Study setting and aim

While EPPs has been successfully rolled out in the daily practice of various developed countries [46–49], they are scarce in the Arab world, with less than 12% of healthcare organizations offering this service [50], the first one was launched in the United Arab Emirates in 2015 [51]. Studies on users’ acceptance of EPP are also sparse in the Middle East region in general [52–54].

This study is based at the American University of Beirut Medical Center- Family Medicine Clinic (AUBMC-FMC), in Lebanon. AUBMC-FMC is a large primary health care center serving mainly AUB faculty, staff, and their families. There are approximately 9469 adult beneficiaries aged 20 years or older, 7.5% are diabetic, and 27% have chronic high blood pressure [55]. Given that EPP is expected to be particularly beneficial for self-management of chronic diseases [56], the focus in this study is on this particular out-patient population.

We conducted this study in 2016–2017, before the launch of the EPP system in November 2018. The “MyChart” EPP system at AUBMC-FMCisa free application accessible via smartphone or computer and linked to the Electronic Medical Record (EMR) using a password. “MyChart” allows patients to schedule appointments, refill prescriptions, directly message their care providers with the option of including photos, access laboratory results, and clinical summaries,. Communication language is English.

This study aims to use the TAM as a framework to quantitatively describe potential users, diabetic and chronic high blood pressure patients and their providers, intention to use and factors influencing the intention to use EPP at AUBMC-FMC. We concurrently test the internal construct validity and the reliability of the TAM framework through a hypothesized structural model.

## Methods

### Study design

We used a cross-sectional survey design and the vignette approach to explore the determinants of users’ acceptance of EPP.

In a typical vignette, respondents are presented with a scenario mimicking a real-life situation and are asked to express their opinions based on this scenario. Vignettes have several advantages over survey questionnaires, including approximating real-life situations, enhancing internal validity and reliability of measurement [57–60], and improving construct validity [61].

The approval of the Ethical Review Committee at AUB was secured before the initiation of this study. The data collection occurred between November 2016 and February 2017.

### Sampling

We targeted patients with diabetes and chronic high blood pressure. To validate an instrument, the recommended respondent-to-item ratio ranges from 5:1 (50 participants for a 10-item questionnaire) to 30:1 [62]. For this study, we used the 15:1 ratio. Further, the recommended minimum sample size for conducting structural equational modeling [52] is 100–150 observations [63–66]. Thus our minimum sample size was 180 patients. All 35 attending physicians and 11 registered nurses actively engaged in primary care services at AUBMC-FMC were targeted.

### Recruitment and data collection

The research assistant identified hypertensive and diabetic patients visiting the clinic, handed the consent form to those who accepted to participate, and interviewed them using the structured survey instrument. Providers were recruited by email, followed by a face-to-face reminder and were prompted to fill the survey online.

We used three different slightly modified versions of the survey for: patient, physician, and nurse. The instrument contained the same vignette, and a questionnaire of two parts. Part A captured demographic characteristics including age, gender, level of education and number of co-morbidities for patients, and years in practice, country of training for providers as well as use of electronic technology in daily lives (independent variables). Part B, contained constructs from the TAM including: intention to use EPP (outcome variable) and service features likely to be used, perceived ease of use, perceived usefulness and social influence (predictor variables). (Appendices A-C).

### Data analysis

Descriptive frequencies for categorical variables or means and standard deviations for ordinal variables were calculated. Exploratory factor analysis (EFA) and structural equational modeling (SEM) [52] were used to test the reliability and validity of the TAM framework. SPSS version 23.0 was used for descriptive statistics, and exploratory factor analysis [67] and AMOS version 21.0 was used to test the hypothesized structural model of the TAM framework [68]. (Appendix D: Hypothesis testing using the TAM framework).

Statistical significance was set at *p* < .05.

EFA and SEM were only carried out for patients, as the sample size of physicians and nurses was too small [65]. Factors explaining variability with eigenvalues smaller than one were not used. Further, we took the factor loading of .5 as a significant cut-off [69].

The STrengthening the Reporting of OBservational studies in Epidemiology (STROBE) guidelines were used to ensure the reporting of this observational study [70] (Appendix E).

## Results

### Participant characteristics

Participants consisted of 199 patients, thus more than the target sample of 180, and 17 providers.

Mean age of patients was 65.1 (*SD* = 13.8, range = 25–92) years, two thirds (62.31%) were males, and 68.84% finished high school level or less; one third (35.68%) reported not using ET in daily activities (Table 1).

**Table 1:** Patients’ demographics, comorbidity and use of technology in daily life

Characteristic	*N (199)*	*%*	*M*	*SD*	*Range*
Age			65.1	13.8	25–92
Sex					
Male	124	62.31			
Female	75	37.69			
Education					
Less than High School	64	32.16			
High School	73	36.68			
College degree	33	16.58			
University degree	29	14.58			
Native Language					
Arabic	194	97.49			
English	5	2.51			
Comorbidities					
1	78	39.2			
2	61	30.65			
More than 2	60	30.15			
Use of electronic technology in daily life*					
Mobile web-based portals	56	28.14			
SMS	125	62.81			
App-based portals	83	41.71			
Social media	73	36.68			
Other e-services (i.e. online banking)	9	4.52			
None of the above	71	35.68			

*Note.* *: frequencies and percentages do not add to 199 and 100 respectively as patients chose more than one option

Mean age of providers was 39.5 (SD = 9.5, range = 27–54) years and 94.12% were females. The mean number of years in practice was 12.4 (SD = 8.1, Range = 0–25) years, and all had one form or another of daily ET activities (Table 2).

**Table 2:** Providers’ demographics, professional and use of technology in daily life

Variable	*N = 17*	*%*	*M*	*SD*	*Range*
Age			39.5	9.6	27–54
Sex					
Male	1	5.88			
Female	16	94.12			
Years in Practice			12.4	8.1	0–25
Country of Training					
Lebanon	11	64.71			
Foreign-trained	1	5.88			
Use of electronic technology in daily life*					
Mobile web-based portals	16	94.12			
SMS	11	64.7			
App-based portals	11	64.7			
Social media	16	94.12			
Other e-services (i.e. online banking)	11	64.7			

*Note.* *: frequencies and percentages do not add to 17 and 100 respectively as providers chose more than one option

### Users acceptance of EPP

**Fig. 2:**
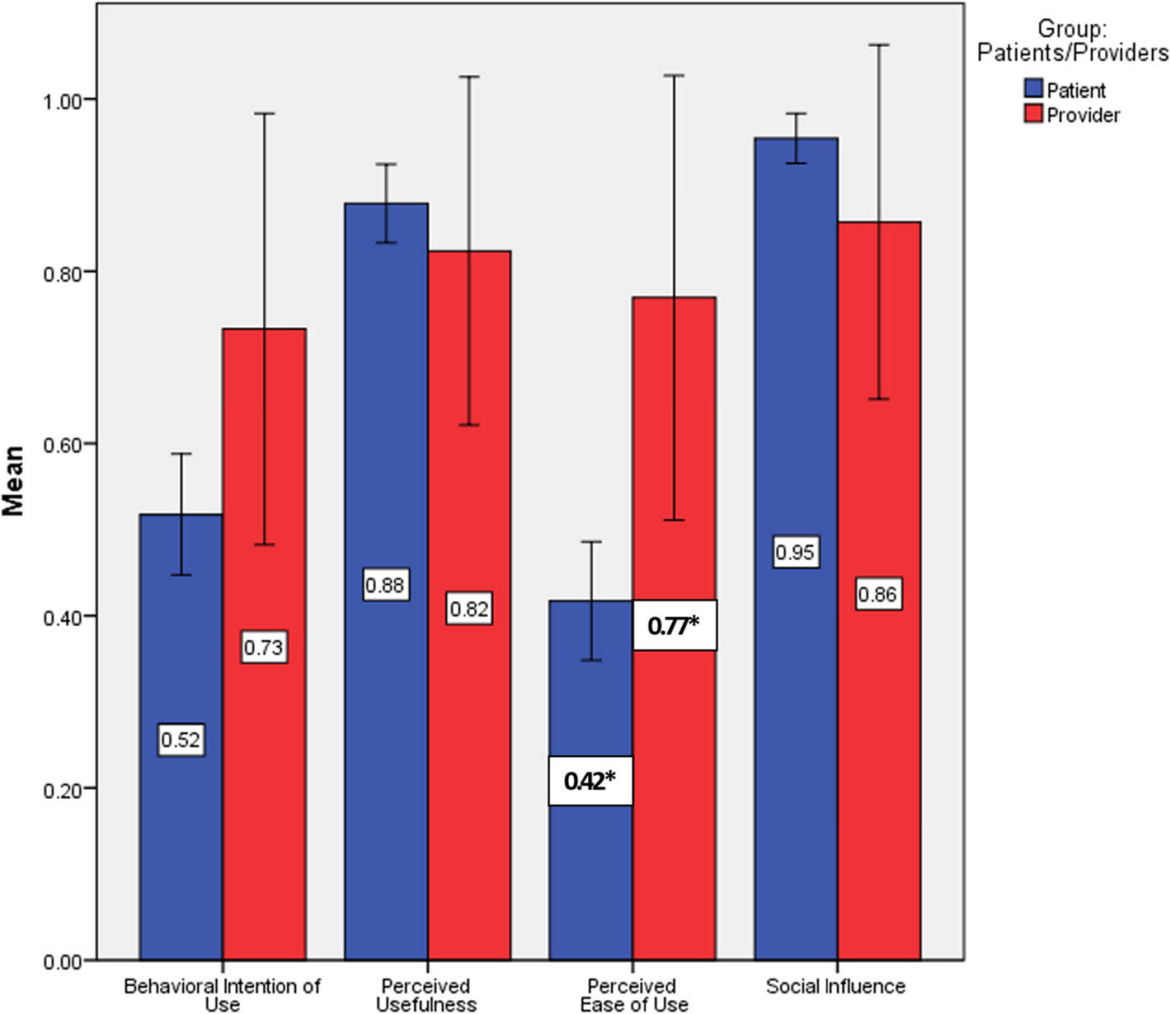
Users’ acceptance of EPP – A contrast between patients and providers. *: Statistically significant different at *p* < .05

Half of the patients intended to use EPP (Fig. 2). When patients were asked about the intention to use various features of the portal, the mean intention to use each feature varied between 3.2 and 3.7 (Table 3). Most patients (88%) perceived the usefulness of the portal. Among the various items that measure the usefulness of the portal use, the importance of saving time ranked highest (*M = 3.6, SD = .9*) and the usefulness of the portal during critical times of the disease ranked least (*M = 2.4, SD = .8*). Only 42% of patients perceived the ease of use of the portal in all aspects of posting information, communicating with the physician, and finding information. Most patients (95%) indicated that social influence will have a positive effect on intention to use while the mean privacy concern was 2.1 (*SD = .9).*

**Table 3:** Patients’ acceptance of EPP based on TAM constructs and probing items

Item	*n*	*%*	*M*	*SD*
Behavioral Intention of Use				
I intend to use EEP to view my medical record.			3.7	1.1
I intend to use EPP to book and reschedule an appointment.			3.7	1.1
I intend to use EPP to refill medication			3.6	1.1
I intend to use EPP to enter my daily weight, daily blood glucose, daily physical exercise activity.			3.2	1.2
I intend to use EPP to receive targeted education from my family physician enabling me to self-manage my disease.			3.7	1.0
Perceived Ease of Use				
Using EPP will be easy for me to understand.			2.5	1.5
It will be easy for me to post information on EPP.			2.5	1.5
I will find it easy to communicate with my primary care physicians using EPP.			2.5	1.5
I will find the information posted by primary care physicians on EPP easy to follow.			2.6	1.5
Perceived Usefulness				
Using EPP will give me greater control over my diabetes/high blood pressure.			3.3	.9
Using EPP will save me time.			3.6	.9
Using EPP will make it easier for me to have a healthier life.			3.4	.9
Using EPP will support me during a critical time of my disease.			2.4	.8
Privacy Concern				
If I use EPP, I will be concerned about my information privacy.			2.1	.9
Social Influence				
If my friends are using EPP and find it worth it, I would use it too.	190	95.48		

Concerning providers, 73% intended to use EPP (Fig. 2), 82.4% intended to post educational material, 70.6% intended to encourage their patients to use the portal for daily recording of weight and blood pressure, and 76.5% intended to encourage patients to use the EPP for administrative tasks, such as booking appointments and refilling medications. As for social influence, 70.59 indicated it has positive effect on intention to use and the mean privacy concern was 3.6 (*SD = 1.1)* (Table 4).

**Table 4:** Providers’ *acceptance of EPP based on TAM constructs and probing items*

Characteristic	*n*	*%*	*M*	*SD*
Behavioral Intention of Use				
I intend to encourage patients to use the appointment booking and medication refill service.	13	76.5		
I intend to encourage my patients to daily record their weight, daily blood pressure and daily blood glucose.	12	70.6		
I will post patient education to assist them self-manage their disease.	14	82.4		
Perceived Ease of Use				
It will be easy for me to post information on EPP.			*3.5*	*0.6*
I will find it easy to communicate with patients using EPP.			*3.2*	*0.9*
Perceived Usefulness				
Using EPP will give me greater control over my patient’s chronic disease.			*4.0*	*0.6*
Using EPP will save me time.			*3.3*	*1.1*
Using EPP will make it easier for me to interact with patients.			*3.6*	*0.9*
Using EPP will support my patients during a critical time of their disease.			*3.5*	*0.8*
Social Influence				
If my colleagues are using EPP and find it worth it, I would use it too.	12	70.59		
Privacy Concern				
I would be concerned about the information privacy of my patients.			3.6	1.1

Figure 2 shows patients vs. healthcare providers’ acceptance of the portal based on TAM’s constructs. Providers (*M =* .77, *SD =* .44) were more likely to perceive the ease of use than patients (*M =* .42, *SD =* .5), <.05, CI [−.623, −.081]; and more privacy concerns (*M* = 3.67, *SD* = 1.11) as compared to patients (*M* = 2.13, *SD* = .9), *p* < .001, CI [−2.16, −.91] (Table 4). There were no statistically significant differences between providers and patients when it comes to intention to use, perceived usefulness of the portal, and social influence.

### Validity, reliability and structural equation model

TAM model reliability revealed an excellent Cronbach Alpha of *α*=.91. To check the internal construct validity, EFA was performed using principal component analysis with Varimax rotation and Kaiser normalization [65]. Three components explained 73.48% of the total variance with an intrinsic value above 1, with a Kaiser-Meyer-Olkin (KMO) sample adequacy of .89. Bartlett’s test of sphericity was statistically significant *χ*^2^(78) = 2401.406, *p* < .001.

Two items (“enter daily weight..” & “receive targeted education ..”) had factor loadings less than .5, thus dropped from the analysis.

First, Behavioral Intention of Use, included three items (Q1, 2 & 3), and explained 14.9% of the variance. It reflected administrative tasks that the portal will facilitate as opposed to the traditional provider encounter. Second, Perceived Ease of Use, included four items (Q4, 5, 6 & 7), and explained 50.74% of the variance. It described the ease of use of the portal to communicate with the provider. Third, Perceived Usefulness, included four items (Q8, 9, 10 & 11), and explained 7.84% of the variance. These items described the use of the portal for better health and better control of chronic diseases and support during critical times (Table 5).

**Table 5:** Exploratory factor analysis

Items	Loadings	Cronbach’s *α*
Behavioral Intention of Use		.815
View my medical record [Q1]	.751	
Book and reschedule an appointment [Q2]	.807	
Refill medication [Q3]	.812	
Perceived Ease of Use		.985
Using EPP will be easy for me to understand [Q4]	.928	
It will be easy for me to post information on EPP [Q5]	.940	
I will find it easy to communicate with my primary care physicians using EPP [Q6]	.941	
I will find the information posted by primary care physicians on EPP easy to follow [Q7]	.921	
Perceived Usefulness		.816
Using EPP will give me greater control over my diabetes/high blood pressure [Q9]	.809	
Using EPP will save me time [Q10]	.507	
Using EPP will make it easier for me to have a healthier life [Q11]	.804	
Using EPP will support me during a critical time of my disease [Q12]	.668	

**Fig. 3:**
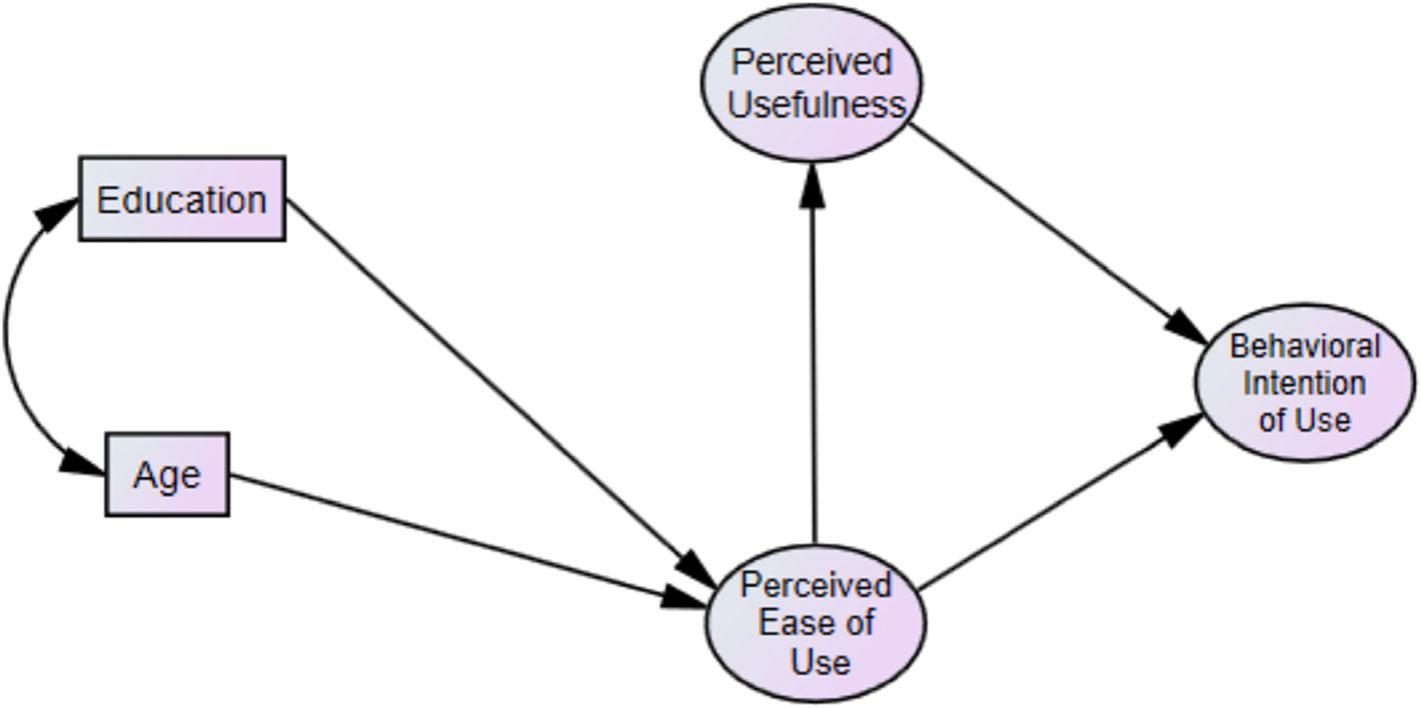
Hypothesized structural model based on the TAM framework

**Fig. 4:**
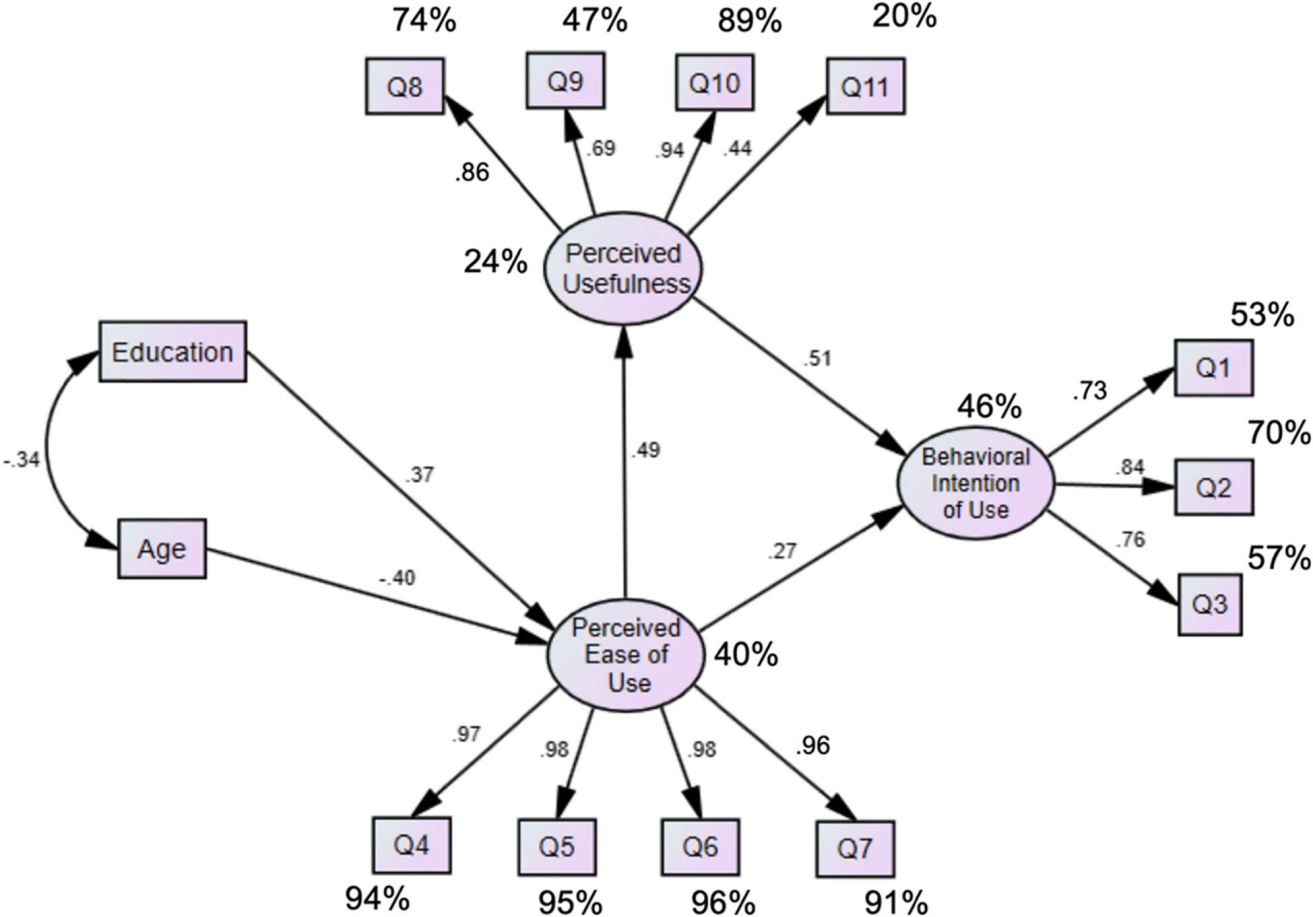
Structural equational model. Percentages indicate squared multiple correlations. All standardized regression coefficients are significant at p < .05

A covariance-based SEM with maximum likelihood was estimated. Figure 3 exhibits the structural model and Fig. 4 shows the analytical results. Fit measures indicated acceptable fit: 푥^2^/푑푓 = 1.9, Tucker Lewis index (TLI) = .97, Comparative Fit index (CFI) = .976, Goodness of Fit Index (GFI) = .918, and Root 153 Mean Square Error of Approximation (RMSEA) = .068. The factors extracted and used in the SEM had also acceptable composite reliabilities (CR) and average variances extracted (AVE): Behavioral Intention of Use had a CR = .83 AVE = .62 . Perceived Usefulness had a CR = .96 and AVE = .87. Perceived Ease of Use had a CR = .8 and AVE = .5. A significant negative correlation between age and education was observed, *r*(197) =  − .339, *p* < .001. As education increased, the perceived ease of use of the EPP increased; and as age increased, using EPP became more difficult. Perceived ease of use increased by .37 standard deviations for every one standard deviation increase in education level, while it decreased by .4 standard deviations for every one standard deviation increase in age. An increase in the perceived ease of use of the EPP predicted an increased perception of the usefulness of the EPP. The standardized regression weight for perceived ease of use on perceived usefulness was .49. Perceived usefulness acted as a mediator between perceived ease of use and behavioral intention of use. Higher reports for both perceived ease of use and perceived usefulness predicted an increase in the behavioral intention of use of the EPP. The standardized regression weights for perceived ease of use and perceived usefulness on the behavioral intention of use were .27 and .51, respectively. Perceived usefulness had more predictive value than perceived ease of use to explain the behavioral intention of use of the EPP.

## Discussion

This study is one of the scarce studies that measure users’ acceptance of EPPs and validate the TAM theoretical model in an Arab country. Given that different cultures react differently to the use of EPP and given the rise in its use, it was important to examine the intention to use to provide decision-makers with an empirical tool that would potentially enhance the actual use. Globally, portal developers, researchers, and practitioners would also benefit from the findings when targeting patients from different cultural backgrounds [71].

We found that providers (physicians and nurses) valued the usefulness of EPP (82%). Our providers were overly optimistic about the perceived usefulness of EPP as compared to a study by Kelly et al. [71], who found that 53% of health care providers valued EPP as a tool to improve patient quality of care. It is possible that this is partially due to the Lebanese culture, which is often rather quick to adopt a positive attitude toward innovation in general [72] and is a novelty-seeking culture, especially among individuals with higher educational attainment [73]. This finding has yet to be tested in actual use as intention and actual use may not be concordant. In fact, in a study conducted by Makarem and Antoun [74] in the same setting, 87.2% of physicians indicated that email was useful for communication, yet only 5.1% used email to communicate with their patients. As for perceived ease of use, 77% of our providers indicated that EPP would be easy to use. This ease of use of EPP and the ability to learn new technology easily by providers can be an important determinant of the actual use [20]. However, further studies are needed to confirm those expectations. Due to the small sample size of providers, we were not able to conduct SEM. Al-Adwan [75] used the TAM model to explore determinants of physician’s adoption of ET in hospitals in Jordan showed that the model explained 64.5% of variance in physician’s behavioral intention.

The intention to use among patients was 52%, lower than 69% [76] and 84.1% [77] reported in the literature. To our knowledge, this is the first study to explore the intention to use the EPP among patients in an Arab region. The lower intention to use is concerning because we expect that actual use will be even less. In a study conducted in Saudi Arabia among diabetic patients, Belcher,Vess, and Johnson [78] explored the use of EPP among diabetic patients and shed light on various limitations associated with actual use among the Arab population. Basic factors such as Internet access, language, and material content may be principal factors for suboptimal use. While the Internet is widely proliferated in most Arab countries, some sub-group of the population may still not have access to the Internet [79]. Patient portal communication is often in English and not all patients may be well versed in the English language [78]. Even among those that are well versed, some may have difficulties understanding medical terms. Wang et al. [80] showed that even top-rated materials often use a language that exceeds the average reading ability. Thus, investing in simplifying messages and tailoring them to the patient’s situation need to be carefully considered during implementation.

We examined the reliability and validity of the TAM model. Taylor and Tod (1995) indicate that a robust model should be able to explain a reasonable proportion of the variance in behavioral intention or use [45]. In this study, we found that the TAM had excellent reliability (Cronbach Alpha of *α*=.91). The validity test (EFA) showed that three components explained 73.48% of the variance, which means that the constructs of the TAM model have a considerably large explanatory power to predict intention to use in an Arab country. However, other models need to be validated in this region such as the Unified Theory of Acceptance and Use of Technology (UTAUT). Further, more contextual factors need to be added to existing models. to explain why technology is accepted or rejected in this specific population.

We found that the perceived usefulness of EPP mostly drives the intent to use. Such a finding is echoed in other studies using the TAM model [81–83]. To bolster EPP use, individuals need to be able to understand the purpose of the technology, which is to provide information when and where it is needed to improve outcomes and patient safety. Hence, organizational efforts focusing not only on promoting the acquisition of the technology but also on marketing the added value of this technology is needed. For example, in our study, patients valued the importance of EPP in controlling their health conditions, promoting their healthy living, and saving them time. Thus, displaying posters in waiting and exam rooms showing patients the usefulness of EPP in facilitating appointment taking, medication refill, and communicating with their physicians can encourage them to use the EPP needs to be considered [84].

We found the perceived ease of use indirectly influenced perceived usefulness. Naturally, When the system is user-friendly and simple, it will likely be successfully used. The human interface with technology matters [85, 86]. For instance, if access to the content of EPP is aesthetically simple, clear and follows a logical process for navigating the system, patients are more likely to use it [87]. Hence, every effort needs to simplify the process of utilization, including training, coaching, and providing continuous support [88, 89].

The digital divide by age and education among our patients were also common observations in studies done in developed countries. For example, in a study carried out on diabetic veterans in North Carolina, USA, lower age, and some college education were more interested in learning how to use EPP [90]. Special considerations need to be taken while addressing older age individuals or those with less educational attainment such as posting educational materials sufficient for a grade 6 reading level, larger fonts, more illustrations, and fewer words can make the EPP more accessible [91–93]. Furthermore, providers need to be proactive by encouraging patients to constantly check their EPP, as this will increase use [94].

Several weaknesses need to be disclosed. We conducted this study with available patients at the AUBMC-FMC, which is a highly recognized organization in Lebanon, capturing patients from higher socioeconomic status or those working in the organization, thus limiting its generalizability to the overall population of Lebanon. Survey completion was voluntary; it is possible that those interested in the topic were more likely to accept our invitation thus possible selection bias. The available small sample of providers prevented us from conducting SEM.

When this study was conducted, the EPP was still under construction. Currently, the system has been launched. Looking forward, longitudinal studies focusing on actual EPP use will be needed. Greenhalgh et al. [95] pointed that likely the optimistic view on the perceived usefulness will be tapered upon the use of the portal, or the perceived ease of use will be influenced with how friendly the system is to patients.

## Conclusions

The most important contribution of this study is that it is the first to report on the acceptability of the patient portal among Lebanese patients and providers, where research is scarce. Further, we were able to successfully and empirically test the predicting factors influencing the intention to use EPP using the TAM model. Based on these findings, we suggest several approaches that can be implemented to encourage the acceptance and utilization of EPP.

### Summary points

What was already known on the topic:

Electronic Patient Portals (EPPs) are one promising form of web-based technology that can be used to mitigate difficulties in booking appointments, renewing medications, and facilitate patients’ interactions with their providers. EPP technology has been successfully rolled out in the daily practice of many developed countries.

The Technology Acceptance Model is a robust and popular framework used to identify factors influencing the adoption of technology. The TAM model has been validated in many countries to assist global developers in increasing the adoption of their technology.

What this study adds:

This study is one of the scarce studies that measure users’ acceptance of EPPs in an Arab country. Half of the patients and 73% of providers intend to use EPP. The structural equation modeling showed two components of TAM explaining the intention to use EPP. Perceived ease of use and perceived usefulness, where the later had more predictive value than the former.

This study also validated the TAM theoretical model among a sample of patients in a high-middle income Arab country, Lebanon.

## Supplementary information

**Additional file 1:** Appendix A Patient vignette Appendix B Patient questionnaire Appendix C Physician vignette

**Additional file 2:** Questionnaire’s items

**Additional file 3:** STROBE Statement—checklist of items that should be included in reports of observational studies

## Data Availability

The datasets used and/or analysed during the current study are available from the corresponding author on reasonable request.

## Abbreviations

AUB: American University of Beirut
AUBMC-FMC: American University of Beirut Medical Center- Family Medicine Clinic
AVE: Average Variances Extracted
CFI: Comparative Fit index
CI: Confidence Interval
CR: Composite reliabilities
EFA: Exploratory factor analysis
EPPs: Electronic Patient Portals
ET: Electronic technology
GFI: Goodness of Fit Index
KMO: Kaiser-Meyer-Olkin
RMSEA: Root Mean Square Error of Approximation
SD: Standard Deviation
SEM: Structural Equation Modeling
STROBE: The STrengthening the Reporting of OBservational studies in Epidemiology
TAM: Technology Acceptance Model
TLI: Tucker Lewis index
