# Supplementary material for: Users’ acceptance of electronic patient portals in Lebanon
Source: BMC Med Inform Decis Mak. 2020 Feb 17;20:31. doi: 10.1186/s12911-020-1047-x (PMC7027116; doi:10.1186/s12911-020-1047-x)
Supplement: Supplementary file 1 — Additional file 1: Appendix A Patient vignette Appendix B Patient questionnaire Appendix C Physician vignette [file 12911_2020_1047_MOESM1_ESM.docx]

Appendix A

Patient vignette

“AUBMC-FMC is intending to introduce a new technology called electronic patient portal enabling you to interact with your primary care physician from the comfort of your home 24 hours a day, 7 days a week. Suppose you are invited to use this service and we provided you with a link to a website where you can enter a password in order to:

1. View your medical record
2. Book and reschedule an appointment and refill your medication
3. Enter your daily weight, daily blood glucose, daily physical exercise activity
4. Receive targeted education from your family physician enabling you to self-manage your disease

Appendix B

Patient questionnaire

Survey ID: ------------------------ Interview Date: ----------------------

Language of interview: ☐ Arabic ☐English

Part A:

1. Gender:

☐ 1- Male

☐ 2- Female

1. Age in years: -----------
2. Education level:

☐ 1- Less than high school

☐ 2- High school

☐ 3- College degree

☐ 4- University degree

1. Co-morbidity:

☐ 1- One

☐ 2- Two

☐ 3- More than two

1. Use of electronic technology in daily lives (please check all that apply):

☐ 1- Mobile web-based portals

☐ 2- SMS

☐ 3- Apps-based portals

☐ 4- Social media such as Facebook and twitter

☐ 5- other e-services such as online banking and online payment.

Part B:

1. I would intend to use the EPP to interact with my primary care physician (Yes/no):------

Why (or why not): -----------------------------------------------------------------

----------------------------------------------------------------------------------------

----------------------------------------------------------------------------------------

1. If I intend to use EPP, I would likely use the following service features (please pick one answer):
   1. View my medical record

☐ 1- Strongly disagree ☐ 2- Disagree ☐ 3- Undecided

☐ 4- Agree ☐ 5- Strongly agree

Why (or why not) : ): -----------------------------------------------------------------

----------------------------------------------------------------------------------------

----------------------------------------------------------------------------------------

- 1. Book and reschedule an appointment

☐ 1- Strongly disagree ☐ 2- Disagree ☐ 3- Undecided

☐ 4- Agree ☐ 5- Strongly agree

Why (or why not) : ): -----------------------------------------------------------------

----------------------------------------------------------------------------------------

----------------------------------------------------------------------------------------

- 1. Refill medication

☐ 1- Strongly disagree ☐ 2- Disagree ☐ 3- Undecided

☐ 4- Agree ☐ 5- Strongly agree

Why (or why not) : ): -----------------------------------------------------------------

----------------------------------------------------------------------------------------

----------------------------------------------------------------------------------------

- 1. Enter your daily weight, daily blood glucose, daily physical exercise activity

☐ 1- Strongly disagree ☐ 2- Disagree ☐ 3- Undecided

☐ 4- Agree ☐ 5- Strongly agree

Why (or why not) : ): -----------------------------------------------------------------

----------------------------------------------------------------------------------------

- 1. Receive targeted education from my family physician enabling me to self-manage my disease

☐ 1- Strongly disagree ☐ 2- Disagree ☐ 3- Undecided

☐ 4- Agree ☐ 5- Strongly agree

Why (or why not) : ): -----------------------------------------------------------------

----------------------------------------------------------------------------------------

----------------------------------------------------------------------------------------

1. EPP will be a useful technology (Yes/no): --------

Because (please pick one answer)

8.1. Using EPP will give me greater control over my diabetes/high blood pressure

☐ 1- Strongly disagree ☐ 2- Disagree ☐ 3- Undecided

☐ 4- Agree ☐ 5- Strongly agree

8.2. Using EPP will save me time

☐ 1- Strongly disagree ☐ 2- Disagree ☐ 3- Undecided

☐ 4- Agree ☐ 5- Strongly agree

8.3. Using EPP will make it easier for me to have a healthier life

☐ 1- Strongly disagree ☐ 2- Disagree ☐ 3- Undecided

☐ 4- Agree ☐ 5- Strongly agree

8.4. Using EPP will support me during a critical time of my disease

☐ 1- Strongly disagree ☐ 2- Disagree ☐ 3- Undecided

☐ 4- Agree ☐ 5- Strongly agree

8.5. Other reasons: -----------------------------------------------------------------

----------------------------------------------------------------------------------------

----------------------------------------------------------------------------------------

1. I will find the EPP easy to use (Yes/no): --------

Because (please pick the best answer)

9.1. Using EPP will be easy for me to understand

☐ 1- Strongly disagree ☐ 2- Disagree ☐ 3- Undecided

☐ 4- Agree ☐ 5- Strongly agree

9.2. It will be easy for me to post information on EPP

☐ 1- Strongly disagree ☐ 2- Disagree ☐ 3- Undecided

☐ 4- Agree ☐ 5- Strongly agree

9.3. I will find it easy to communicate with my primary care physicians using EPP

☐ 1- Strongly disagree ☐ 2- Disagree ☐ 3- Undecided

☐ 4- Agree ☐ 5- Strongly agree

9.4. I will find the information posted by primary care physicians on EPP easy to follow

☐ 1- Strongly disagree ☐ 2- Disagree ☐ 3- Undecided

☐ 4- Agree ☐ 5- Strongly agree

9.5. Other reasons: -----------------------------------------------------------------

----------------------------------------------------------------------------------------

----------------------------------------------------------------------------------------

1. If my friends are using EPP and find it worth it, so I would use it too: (Yes/no):---

Why (or why not) : -----------------------------------------------------------------

----------------------------------------------------------------------------------------

----------------------------------------------------------------------------------------

11- If I use EPP, I will be concerned about my information privacy:

☐ 1- Strongly disagree ☐ 2- Disagree ☐ 3- Undecided

☐ 4- Agree ☐ 5- Strongly agree

What types of privacy issues are you concerned about: ----------------------

----------------------------------------------------------------------------------------

----------------------------------------------------------------------------------------

Appendix B

**Physician vignette**

“AUBMC-FMC is introducing a new technology called electronic patient portal enabling patients to interact with primary care physician from the comfort of their home 24 hours a day, 7 days a week. Suppose you are invited to use this service for your patients. The system will allow patients to login to a website using a password, and this system is tethered to their medical records allowing patients to view a summary of their clinical information in addition they can book and reschedule an appointment and refill medication, enter their daily weight, blood glucose, physical exercise activity, and receive targeted educational materials”.

**Physician questionnaire**

Survey ID: ------------------------ Interview Date: ----------------------

Part A:

1. Gender:

☐ 1- Male

☐ 2- Female

1. Age in years: -----------
2. Years in practice:---------
3. Country of training:

☐ 1- Lebanon

☐ 2- North America

☐ 3- Europe

☐ 4- Other, please specify: -----------------------------

1. Use of electronic technology in daily lives (please check all that apply):

☐ 1- Mobile web-based portals

☐ 2- SMS

☐ 3- Apps-based portals

☐ 4- Social media such as facebook and twitter

☐ 5- other e-services such as online banking and online payment.

Part B:

1. I would intend to use the EPP to interact with my patients (Yes/No):------

Why (or why not): -----------------------------------------------------------------

----------------------------------------------------------------------------------------

----------------------------------------------------------------------------------------

1. EPP is a useful technology (Yes/No): --------

Because (please pick one answer)

7.1. Using EPP will give me greater control over my patient’s chronic disease

☐ 1- Strongly disagree☐ 2- Disagree ☐ 3- Undecided

☐ 4- Agree ☐ 5- Strongly agree

7.2. Using EPP will save me time

☐ 1- Strongly disagree ☐ 2- Disagree ☐ 3- Undecided

☐ 4- Agree ☐ 5- Strongly agree

7.3. Using EPP will make it easier for me to interact with my patients

☐ 1- Strongly disagree ☐ 2- Disagree ☐ 3- Undecided

☐ 4- Agree ☐ 5- Strongly agree

7.4. Using EPP will help me support my patients during a critical time of their disease

☐ 1- Strongly disagree ☐ 2- Disagree ☐ 3- Undecided

☐ 4- Agree ☐ 5- Strongly agree

7.5. Other reasons: -----------------------------------------------------------------

----------------------------------------------------------------------------------------

----------------------------------------------------------------------------------------

1. I will find the EPP easy to use (Yes/no): --------

Because (please the best answer)

8.1. It will be easy for me to post information on EPP

☐ 1- Strongly disagree ☐ 2- Disagree ☐ 3- Undecided

☐ 4- Agree ☐ 5- Strongly agree

8.2. I will find it easy to communicate back and forth with my patients using EPP

☐ 1- Strongly disagree ☐ 2- Disagree ☐ 3- Undecided

☐ 4- Agree ☐ 5- Strongly agree

8.3. Other reasons: -----------------------------------------------------------------

----------------------------------------------------------------------------------------

----------------------------------------------------------------------------------------

1. If my colleagues are using EPP and find it worth it, so I would use it too: (Yes/No):------

Why (or why not): -----------------------------------------------------------------

----------------------------------------------------------------------------------------

----------------------------------------------------------------------------------------

1. I will intend to encourage my patients to use the appointment booking and medication refill service (Yes/no): --------

Why (or why not): -----------------------------------------------------------------

----------------------------------------------------------------------------------------

----------------------------------------------------------------------------------------

1. I will intend to encourage my patients to daily record their weight, blood pressure and blood glucose (Yes/no): --------

Why (or why not): -----------------------------------------------------------------

----------------------------------------------------------------------------------------

----------------------------------------------------------------------------------------

1. I will post patient education to assist them self-manage their disease

(Yes/no): --------

Why (or why not): -----------------------------------------------------------------

----------------------------------------------------------------------------------------

----------------------------------------------------------------------------------------

1. I would be concerned about the information privacy of my patients

☐ 1- Strongly disagree ☐ 2- Disagree ☐ 3- Undecided

☐ 4- Agree ☐ 5- Strongly agree

What types of privacy issues are you concerned about: ----------------------

----------------------------------------------------------------------------------------

----------------------------------------------------------------------------------------

Appendix C

**Nurse Vignette**

“AUBMC-FMC is introducing a new technology called electronic patient portal enabling patients to interact with primary care physician from the comfort of their home 24 hours a day, 7 days a week. Suppose you are invited to use this service for your patients. The system will allow patients to login to a website using a password, and this system is tethered to their medical records allowing patients to view a summary of their clinical information in addition they can book and reschedule an appointment and refill medication, enter their daily weight, blood glucose, physical exercise activity, and receive targeted educational materials”

**Nurse Questionnaire**

Survey ID: ------------------------ Interview Date: ----------------------

Part A:

1. Gender:

☐ 1- Male

☐ 2- Female

1. Age in years: -----------
2. Years in practice:---------
3. Country of training:

☐ 1- Lebanon

☐ 2- North America

☐ 3- Europe

☐ 4- Other, please specify: -----------------------------

1. Use of electronic technology in daily lives (please check all that apply):

☐ 1- Mobile web-based portals

☐ 2- SMS

☐ 3- Apps-based portals

☐ 4- Social media such as facebook and twitter

☐ 5- other e-services such as online banking and online payment.

Part B:

1. I would intend to use the EPP to interact with my patients (Yes/No):------

Why (or why not): -----------------------------------------------------------------

----------------------------------------------------------------------------------------

----------------------------------------------------------------------------------------

1. EPP is a useful technology (Yes/No): --------

Because (please pick one answer)

7.1. Using EPP will give me greater control over my patient’s chronic disease

☐ 1- Strongly disagree☐ 2- Disagree ☐ 3- Undecided ☐ 4- Agree ☐ 5- Strongly agree

7.2. Using EPP will save me time

☐ 1- Strongly disagree ☐ 2- Disagree ☐ 3- Undecided ☐ 4- Agree ☐ 5- Strongly agree

7.3. Using EPP will make it easier for me to interact with my patients

☐ 1- Strongly disagree ☐ 2- Disagree ☐ 3- Undecided ☐ 4- Agree ☐ 5- Strongly agree

7.4. Using EPP will help me support my patients during a critical time of their disease

☐ 1- Strongly disagree ☐ 2- Disagree ☐ 3- Undecided ☐ 4- Agree ☐ 5- Strongly agree

7.5. Other reasons: -----------------------------------------------------------------

----------------------------------------------------------------------------------------

----------------------------------------------------------------------------------------

1. I will find the EPP easy to use (Yes/no): --------

Because (please the best answer)

8.1. It will be easy for me to post information on EPP

☐ 1- Strongly disagree ☐ 2- Disagree ☐ 3- Undecided ☐ 4- Agree ☐ 5- Strongly agree

8.2. I will find it easy to communicate back and forth with my patients using EPP

☐ 1- Strongly disagree ☐ 2- Disagree ☐ 3- Undecided ☐ 4- Agree ☐ 5- Strongly agree

8.3. Other reasons: -----------------------------------------------------------------

----------------------------------------------------------------------------------------

----------------------------------------------------------------------------------------

1. If my colleagues are using EPP and find it worth it, so I would use it too: (Yes/No):------

Why (or why not): -----------------------------------------------------------------

----------------------------------------------------------------------------------------

----------------------------------------------------------------------------------------

1. I will intend to encourage my patients to use the appointment booking and medication refill service (Yes/no): --------

Why (or why not): -----------------------------------------------------------------

----------------------------------------------------------------------------------------

----------------------------------------------------------------------------------------

1. I will intend to encourage my patients to daily record their weight, blood pressure and blood glucose (Yes/no): --------

Why (or why not): -----------------------------------------------------------------

----------------------------------------------------------------------------------------

----------------------------------------------------------------------------------------

1. I will post patient education to assist them self-manage their disease

(Yes/no): --------

Why (or why not): -----------------------------------------------------------------

----------------------------------------------------------------------------------------

----------------------------------------------------------------------------------------

1. I would be concerned about the information privacy of my patients

☐ 1- Strongly disagree ☐ 2- Disagree ☐ 3- Undecided ☐ 4- Agree ☐ 5- Strongly agree

What types of privacy issues are you concerned about: ----------------------

----------------------------------------------------------------------------------------

----------------------------------------------------------------------------------------
