# Supplementary material for: Users’ acceptance of electronic patient portals in Lebanon
Source: BMC Med Inform Decis Mak. 2020 Feb 17;20:31. doi: 10.1186/s12911-020-1047-x (PMC7027116; doi:10.1186/s12911-020-1047-x)
Supplement: Supplementary file 2 — Additional file 2: Questionnaire’s items [file 12911_2020_1047_MOESM2_ESM.docx]

Questionnaire’s items

| The scales’ items were measured on a five-point Likert scale, ranging from “strongly disagree” (1) to “strongly agree” (5) unless indicated otherwise | | |
| --- | --- | --- |
| **Construct** | **Items** | **Hypothesis** |
| EPP will be a useful technology  (Yes/No) | Using EPP will give me greater control over my diabetes/high BP  Using EPP will save me time  Using EPP will make it easier for me to have a healthier life  Using EPP will support me during a critical time of my disease | H01: Perceived usefulness will have a positive effect on intention to use |
| EPP will be easy to use  EU (Yes/No) | Using EPP will be easy for me to understand  It will be easy for me to post information on EPP  I will find it easy to communicate with my primary care physicians using EPP | H02: Perceived ease to use will have a positive effect on intention to use |
|  | I will find the information posted by primary care physicians on EPP easy to follow |  |
| Subjective and social  norm SI | If my friends are using EPP and find it worth it, so I would use it too (Yes/No)  If I use EPP, I will be concerned about my information privacy | H03: Social influence will have a positive effect on intention to use |
| Behavioral Intention | I intend to use EHR Portals (Yes/No) | Descriptive presentations |
| Age |  | H04: Age will moderate the effect of ease to use on intention to use so that the effect will be stronger for younger age |
| Age |  | H05: Age will moderate the effect of useful use on intention to use so that the effect will be stronger for younger age |
| Sex |  | H06: will moderate the effect of ease to use on intention to use so that the effect will be stronger for female |
| Gender |  | H07: will moderate the effect of useful use on intention to use so that the effect will be stronger for women |
| Co-morbidity (ordinal: 1, 2, more than 2) |  | H08: co-morbidity will moderate the effect of ease to use on intention to use so that the effect will be stronger for higher-comorbidity |
| Co-morbidity |  | H09: co-morbidity will moderate the effect of useful use on intention to use so that the effect will be stronger for higher-comorbidity |
| Facilitating Conditions | Use of electronic technology in daily lives (nominal variable) | H10: Facilitating conditions will have a positive effect on intention to use so that the effect will be stronger with higher use of technology |
